# Incidence of suicidal ideation in a cohort of civil servants during the COVID-19 pandemic in Brazil: insights from the ELSA-Brasil Study

**DOI:** 10.47626/2237-6089-2023-0701

**Published:** 2024-10-28

**Authors:** Pedro Bacchi, Paulo Suen, Daniel Fatori, Lais B. Razza, Leonardo Afonso, Izio Klein, Beatriz Cavendish, Marina L. Moreno, Itamar S. Santos, Isabela Benseñor, Paulo Lotufo, André R Brunoni

**Affiliations:** 1 Universidade de São Paulo Instituto de Psiquiatria Faculdade de Medicina São Paulo SP Brazil Laboratório de Neurociências (LIM-27), Departamento de Psiquiatria, Instituto de Psiquiatria, Faculdade de Medicina, Universidade de São Paulo (USP), São Paulo, SP, Brazil.; 2 Faculdade de Medicina São Paulo SP Brazil Faculdade de Medicina, USP, São Paulo, SP, Brazil.; 3 Faculdade de Medicina Departamento de Clínica Médica São Paulo SP Brazil Departamento de Clínica Médica, Faculdade de Medicina, USP, São Paulo, SP, Brazil.; 4 Hospital Universitário Centro de Pesquisas Clínicas e Epidemiológicas São Paulo SP Brazil Centro de Pesquisas Clínicas e Epidemiológicas, Hospital Universitário, USP, São Paulo, SP, Brazil.

**Keywords:** Suicidal ideation, suicide, Covid-19, pandemic, Brazil

## Abstract

**Objective:**

This study investigated the incidence of suicidal ideation and its associated risk factors in the state of São Paulo in the Brazilian Longitudinal Study of Adult Health (Estudo Longitudinal de Saúde do Adulto [ELSA-Brasil]) cohort during the coronavirus disease 2019 (COVID-19) pandemic.

**Methods:**

In a pre-pandemic ELSA-Brasil onsite assessment in 2016-2018 (wave 3) and a pandemic online assessment in May-July 2020 (wave COVID), we assessed suicidal ideation using the Clinical Interview Schedule-Revised (CIS-R). Single and multi predictor logistic regressions were performed using sociodemographic characteristics, household financial impact during the pandemic, presence of previous chronic diseases, alcohol abuse, adverse childhood experiences (ACE), living alone, and previous common mental disorders (CMD) as predictors. Incidence of suicidal ideation was used as outcome.

**Results:**

Out of 4,191 participants in wave 3, 2,117 (50.5%) also answered the COVID wave. There was a threefold increase in suicide ideation, from 34 (1.8%) to 104 (5.6%) participants. In multiple predictor models, we found that previous CMD (odds ratio [OR] 7.17; 95% confidence interval [95%CI] 4.43 - 11.58) and ACE (OR 1.72; 95%CI 1.09 - 2.72) increased the odds of incident suicidal ideation. The sociodemographic predictors female sex, younger age, and low income were significant risk factors in the single predictor models only.

**Conclusion:**

These findings underscore the importance of monitoring and supporting individuals who suffered ACE and have a history of mental health disorders. This is especially critical in times of heightened societal stress, such as the COVID-19 pandemic.

## Introduction

In early 2020, the global spread of coronavirus disease 2019 (COVID-19) led to significant lifestyle shifts,^1^ which were hypothesized to contribute to a substantial rise in mental disorders and suicide rates.^2^ However, large cohort studies and meta-analyses involving over 72,000 participants have shown only minor increases in mental disorders during the first 2 years of the pandemic in both high and low income countries.^3–6^ In Brazil, for instance, a cohort study using data from The Brazilian Longitudinal Study of Adult Health (Estudo Longitudinal de Saúde do Adulto [ELSA-Brasil]) found no worsening in depressive and anxiety disorders.^7^ Nevertheless, there are fewer studies from low to middle income countries (LMIC), which are home to 85% of the world's population, account for 75% of global suicides,^8^ and face dire socioeconomic and public health challenges.

Regarding suicide rate, a recent meta-analysis with 45 studies demonstrated that the rate of suicides remained mostly unchanged when comparing pre-pandemic (before 2020) to peripandemic (2020-2022) periods, with the vast majority of the studies including samples from high-income countries and none from Brazil.^9^ Interestingly, previous studies report that suicide rates decreased in Brazil^10^ and exhibited no important changes in other LMICs during the first year of the pandemic (2020).^11^ The phenotypes across the suicide spectrum include suicidal ideation, suicide attempt, and death by suicide. Suicidal ideation comprises any thoughts about ending one's own life, which may be active, with a clear plan for suicide, or passive, with thoughts about wishing to die.^12^ Suicide ideation occurs in depressive states of different psychopathologies, and its transition to suicide attempt can be facilitated by comorbidity with impulse disorders or conditions that increase distress.^13^

Suicidal ideation is believed to originate from a complex interplay of biological, psychological, and social factors.^14^ Sociodemographic factors including age, gender, and socioeconomic status are considered important risk factors.^15^ Furthermore, individuals exposed to adverse childhood experiences (ACE), both direct (e.g., abuse and neglect) and indirect (e.g., parental conflict, substance abuse, or mental illness in the family), often grapple with more physical and mental health issues in adulthood, including a higher predisposition towards suicide ideation.^16^ Other environmental and behavioral factors, such as social isolation, alcohol abuse, and financial difficulties further increase susceptibility to suicidal ideation.^15^ Considering the extensive impact of the COVID-19 pandemic on many of these risk determinants and potential unknown mediators, there is significant scope for understanding the incidence of suicidal ideation during this period.

Therefore, this study investigated the incidence of suicidal ideation in the ELSA-Brasil civil servants cohort from the last assessment prior to the pandemic, which occurred during 2016-2018, to the early pandemic period during the first semester of 2020. We evaluated whether sociodemographic characteristics, the financial household impact caused by the pandemic, presence of previous chronic diseases, alcohol abuse, ACE, living alone, and previous common mental disorders (CMD) might be predictors of suicidal ideation during the pandemic. We hypothesized that women, individuals whose families suffered financial losses, individuals presenting chronic diseases, individuals who had ACE, and individuals with previous CMD would have a higher incidence of suicidal ideation.

## Methods

### Study design and participants

ELSA-Brasil is a cohort of 15,105 civil servants aged 35 to 74 years-old evaluated since 2008-2010 in six Brazilian state capitals (São Paulo, Rio de Janeiro, Minas Gerais, Espírito Santo, Bahia, and Rio Grande do Sul). The study aims to investigate the development and progression of clinical and subclinical chronic diseases in a population from a low-middle income country. The baseline assessment was conducted in 2008-2010 (first wave) and included 15,105 participants aged between 35 and 74 years, followed by two posterior waves in 2012-2014 (second wave), and 2016-2018 (third wave). Each wave consisted of comprehensive onsite assessments comprising clinical interviews, psychiatric assessments, medical examinations, and laboratory tests, collecting information on sociodemographic variables, clinical history, family history of diseases, lifestyle factors, anthropometric measurements, and biomarkers.^17,18^

During the onset of the COVID-19 pandemic, from May to July of 2020, all participants from the São Paulo research center (active or retired public servants from the University of São Paulo [USP]; n = 4,191) who had responded to the third wave were invited to respond to an online assessment to investigate psychiatric disorders and symptoms (which was called the COVID wave). More details and initial findings can be found in our previous papers.^7^

Our study was approved by the local ethics committee at the USP university hospital. All participants provided electronic informed consent. All procedures contributing to this research comply with the ethical standards of the relevant national and institutional committees on human experimentation and with the Helsinki Declaration of 1975, as revised in 2008.

In the course of our study, participants expressing suicidal ideation or self-reported poor mental health were referred for an online psychiatric consultation. To address this need promptly and effectively, we assembled a dedicated team of psychiatrists who were readily available for online interactions, ensuring timely intervention and support for those individuals.

### Psychiatric disorders and assessment of symptoms

The Clinical Interview Schedule-Revised (CIS-R) was applied at both wave 3 and wave COVID. It includes assessment of 14 symptoms and 13 psychiatric disorders based on the International Classification of Disease, 10th revision (ICD-10). The CIS-R domains are somatic complaints, fatigue, concentration and forgetfulness, sleep disturbance, irritability, worry about physical health, depression, depression ideas, worry, anxiety, phobias, panic attacks, compulsions, and obsessions. Scores for each section range from 0 to 4 (except for the score for depressive ideas, which ranges from 0 to 5); therefore, the total score ranges from 0 to 57. A diagnosis of CMD is operationally defined based on this score (CIS-R > 11).^19^

The CIS-R assesses the presence of suicidal ideation with the following question: "in the last 7 days, did you consider that life is not worth living?", to which the participant can answer yes or no. This assessment has been used in another study, in the United Kingdom, during the pandemic.^20^ We defined incident suicidal ideation as present if a participant answered this question in the affirmative at wave COVID but not at wave 3. This variable was used as the primary outcome of this study.

### Sociodemographic characteristics

Sociodemographic characteristics were obtained in the wave 3 assessment and included age, sex, educational level (college degree or below college degree), total household income, and self-reported race (white or non-white) (Table 1).

**Table 1:** Characteristics of the sample

Variable	Suicidal ideation incidence (n = 89)	No incidence (n = 1,730)	Total (n = 1,819)	p-value*
Women	63 (70.8)	1002 (57.9)	1065 (58.5)	0.016
Age, mean (SD)	58.5 (7.4)	60.7 (8.3)	60.6 (8.3)	< 0.001
White	56 (62.9)	1177 (68.0)	1233(67.8)	0.31
College degree	47 (52.8)	1083 (62.6)	1130 (62.1)	0.06
High income†	32 (35.9)	863 (49.9)	895 (49.2)	0.01
Common mental disorder‡	59 (66.2)	321 (18.6)	380 (20.9)	< 0.001
Financial impact§	25 (28.1)	334 (19.31)	359 (19.7)	0.04
Previous chronic disease	56 (62.9)	873 (50.5)	929 (51.0)	0.02
Lives alone	18 (20.2)	285 (16.5)	303 (16.7)	0.35
Alcohol excess	4 (4.5)	65 (3.7)	69 (3.8)	0.72
Childhood adversity||	52 (58.4)	649 (37.5)	701 (38.5)	< 0.001

Data presented as n (%).

SD = standard deviation.

* *t* test for age and chi-square for binary variables.

† Household income superior to the median of the sample.

‡ Had a common mental disorder diagnosis at wave 3 (2016-2018).

§ Household income fell more than 50% during the pandemic.

|| Had at least one adverse childhood experience.

### Exposures

Alcohol abuse was defined as > 1 dose/day for women and > 2 doses/day for men during a given week, at wave 3. Previous chronic disorder was defined as self-reported presence of one or more of diabetes, high blood pressure, coronary heart disease, stroke, asthma, chronic bronchitis, or other chronic conditions at wave COVID. More details regarding the assessment of exposures can be found elsewhere.^7^

We did not assess loneliness symptoms at wave COVID. Instead, we assessed whether the participant was living alone at the time of wave COVID.

At wave 3, participants were asked about ACE: (a) lived with someone who abused drugs/alcohol/medicines; (b) lived with someone who was arrested/convicted; (c) lived with someone with depression or other mental disorder; (d) parents separated/divorced; (e) parents or guardians died before subject was 14 years old; (f) worked during childhood. Participants who reported at least one event were classified as having experienced an ACE.

Participants were asked whether their household finances had suffered an impact during the pandemic. If the household's monthly income was less than 50% of its pre-pandemic level, we defined the household as having suffered a financial impact.

### Statistical analyses

Statistical analyses were conducted using R Studio 4.3.1 and Stata. We described demographic data using frequencies, and comparisons between groups were performed using the chi-square test for binary variables and the *t* test for continuous variables. An alpha threshold of 0.05 was used as the significance level.

Logistic regression models were estimated with the Stata command *logistic* to investigate associations between exposures and incident suicidal ideation. We excluded participants who presented suicidal ideation before the pandemic, thus including only incident cases (no suicidal ideation at wave 3 and suicidal ideation at wave COVID) and absent cases (no suicidal ideation at either wave).

First, we performed single predictor logistic regressions using the suicidal ideation incidence as a binary outcome and the following variables as predictors: age, sex, baseline income, having a college degree, being white, having previous chronic diseases, having had household financial impact during the pandemic, alcohol abuse, ACE, living alone and previous CMD. We then performed a multiple predictor logistic regression using the same outcome and excluding predictors that had not had a significant result in the single predictor analysis. We adjusted the multiple predictor model for sociodemographic variables, so age, college degree, race, and baseline income were not excluded.

## Results

### Participants

Out of 4,191 eligible participants who had answered wave 3, 2,117 (50.5%) answered at least one assessment at wave COVID, and 1,853 (44.2%) answered both the comprehensive questionnaire and the CIS-R assessments. Compared to non-respondents, the subset of participants who responded to the wave COVID assessment had a significantly higher percentage of women, were younger, with a higher educational level, and had lower rates of psychiatric symptoms and diagnoses (supplementary Table S1).

Among the 1,853 participants who answered both waves, 34 (1.8%) presented suicidal ideation at wave 3, and 104 (5.6%) at wave COVID. We observed persistence (present in both waves) of suicidal ideation in 15 (0.8%) participants, remission (present only at wave 3) in 19 (1%) participants, and incidence (present only at wave COVID) in 89 (4.8%) participants.

**Figure 1:**
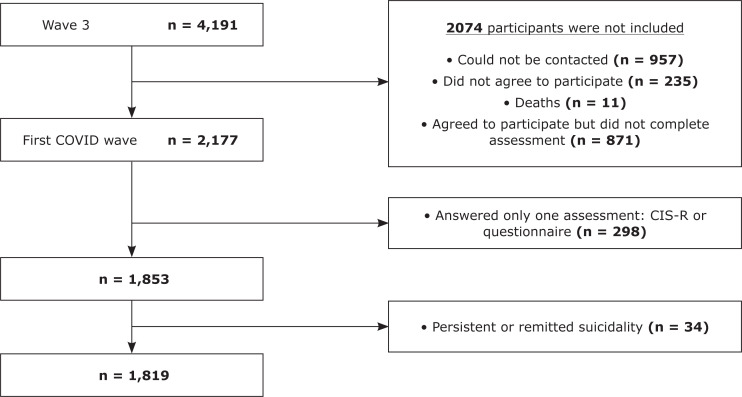
Flow diagram of the present study. CIS-R = Clinical Interview Schedule-Revised; COVID = coronavirus disease.

Since our analysis excluded participants with suicidal ideation at wave 3, a total of 1,819 participants were included. Non-inclusion reasons were unwillingness to participate, impossibility of making contact, not answering the survey completely, and deaths (Figure 1). We included participants who answered the comprehensive questionnaire and provided CIS-R data at wave 3 and wave COVID. Characteristics of the sample are described in Table 1.

In the single predictor model, we found that women (odds ratio [OR] 1.76; 95% confidence interval [95%CI] 1.10-2.80), individuals with previous CMD (OR 8.63; 95%CI 5.47-13.62), chronic diseases (OR 1.66; 95%CI 1.07-2.59), or household financial impact (OR 1.63; 95%CI 1.01-2.63), individuals living alone (OR 1.28; 95%CI 0.75-2.10), and individuals with ACE (OR 2.34; 95%CI 1.51-3.60) had significantly increased odds of incident suicidal ideation, while older age (OR 0.96; 95%CI 0.94-0.99) and high income (OR 0.56; 95%CI 0.36-0.88) significantly decreased those odds. Race, alcohol abuse, and formal education did not significantly alter the odds of incident suicidal ideation.

In the multiple predictor model, we found that previous CMD (OR 3.62; 95%CI 2.18-6.01) and ACE (OR 1.72; 95%CI 1.09-2.72) significantly increased the odds of incident suicidal ideation. In this model, sex, age, race, formal education, previous chronic disease, living alone, baseline high income, alcohol abuse, and household financial impact were not significant (Table 2).

**Table 2:** Predictor models for incidence of suicidal ideation

	Simple model*		Multi-predictor model†	
	OR	95%CI	OR	95%CI
Sex (female)	1.76	1.10-2.80‡	1.16	0.71-1.91
Age	0.96	0.94-0.99‡	0.98	0.95-1.02
White	0.79	0.51-1.24	1.18	0.77-1.91
College degree	0.67	0.43-1.02	0.82	0.49-1.38
High income§	0.56	0.36-0.88‡	0.99	0.55-1.76
Common mental disorder||	8.63	5.47-13.62¶	7.17	4.43-11.58¶
Financial impact**	1.63	1.01-2.63‡	1.32	0.79-2.18
Chronic diseases	1.66	1.07-2.59‡	1.53	0.96-2.42
Alcohol abuse	1.20	0.43-3.38	-	-
Childhood adversity††	2.34	1.51-3.60¶	1.72	1.09-2.72‡
Living alone	1.28	0.75-2.10	-	-

95%CI = 95% confidence interval; OR = odds ratio.

* Single predictor logistic regression model with suicidal ideation incidence as outcome;

† Multiple predictor logistic regression model with the same outcome, including only significant predictors from the single predictor models and adjusted for sociodemographic variables;

‡ p < 0.05;

§ Household income superior to the median of the sample;

|| Had a common mental disorder diagnosis at wave 3 (2016-2018);

¶ p < 0.001;

** Household income fell more than 50% during pandemic;

†† had at least one adverse childhood experience.

## Discussion

This study investigated the incidence of suicidal ideation among participants of the ELSA-Brasil cohort from São Paulo state, comparing the pre pandemic third wave (2016-2018) with the peripandemic first COVID wave (2020). There was a threefold increase in suicidal ideation from before the pandemic to during the pandemic in our sample. We found that ACE and previous CMD were relevant risk factors for incidence of suicidal ideation during the pandemic. Female sex, younger age, and low income were sociodemographic predictors associated with suicidal ideation incidence in single predictor models. No significant associations were found with race, formal education, household financial impact caused by the pandemic, presence of previous chronic diseases, alcohol abuse, or living alone.

The findings of this study underscore the impact of previous mental disorders on the incidence of suicidal ideation during the first year of COVID-19. The OR of this exposure was large (7.7) and supports the findings of previous studies.^15^ Furthermore, a meta-analysis that included 19 studies and more than 11 thousand participants found that previous mental health, quarantine, loneliness, and exhaustion were the main risk factors for suicidal ideation during the pandemic.^14^ A Bayesian network analysis study performed in the United Kingdom and Austria revealed that depressive symptoms and anxiety symptoms were among the most important risk factors for suicidal ideation during the pandemic.^21^ A longitudinal study with 2,441 US military veterans found that participants with preexisting loneliness, psychiatric distress, and lower purpose in life were at heightened risk of developing new-onset suicidal ideation and suicide planning during the pandemic.^22^ The lifestyle and social changes observed during lockdowns may have amplified the influence of mental health disorders on suicidal ideation. Instead of using specific diagnoses of anxiety or depressive disorders as exposures, we opted to use the construct of CMD, which can be assessed with the CIS-R. Taking into account the intersection of hopelessness and suicidal ideation on the construct of depression diagnoses, to avoid excessive collinearity with the outcome, the CMD construct seemed a broader exposure to predict suicidal ideation.

The results suggest that individuals who had ACE were at increased risk for suicidal ideation incidence during the pandemic. Unfortunately, our pre pandemic assessment did not include questions regarding physical and sexual abuse, which are considered important risk factors for suicidal ideation,^23^ but did include experiences that can be considered exposures to household dysfunction, such as emotional and physical neglect, household substance abuse, household mental illness, and parental separation or divorce. Household dysfunctions may be especially important forms of ACE and are associated with the leading causes of death in adults, including suicide.^24,25^ A study with 55,299 respondents from nationally representative samples found that ACE have the strongest associations with suicide attempts in childhood, decreasing during teen years and young adulthood, and increasing in later adulthood.^25^ The mechanisms underlying this association in older age, which matches our sample, are multifactorial and have biological, psychiatric, clinical and psychosocial dimensions. Older adults in general have improved emotion regulation skills and hence higher levels of affective well-being. On the other hand, individuals who had ACE present maladaptive cognitive styles and impaired coping and problem solving skills and seem to be even more reactive to late-life stressors.^26^ Difficulties related to the pandemic may have had stronger impacts in older individuals who had ACE, since in this age group, late-life stressors frequently precede suicidal behaviors.^27^

The association between baseline income and the incidence of suicidal ideation observed in our study underscores the potential vulnerability of individuals with lower income levels, even within a relatively financially secure cohort of civil servants. Job loss or financial problems are known risk factors for suicide.^28^ Unlike baseline income, the financial impact on the household experienced during the early phase of the pandemic did not significantly correlate with suicidal ideation in our sample. This discrepancy might be attributable to different factors. Firstly, the assessment of financial impact occurred relatively close to the onset of the pandemic, potentially before the full economic repercussions could be felt by individuals and their families. Secondly, even if the household suffered financial losses, the inherent financial stability of having at least one civil servant in the family might have alleviated the distress associated with finances. Our findings suggest that long-standing financial status, represented by baseline income, might have a more enduring influence on mental health outcomes such as suicidal ideation, compared to short-term financial disruptions.

Although alcohol abuse is a well-established risk factor for suicide,^29^ it was not associated with an increased risk of suicide ideation in our study. We defined alcohol abuse as the consumption of more than one drink per day for women and more than two drinks per day for men over a given week, aligning with common thresholds.^30^ However, this definition did not differentiate among domains of alcohol use, such as binge drinking and dependence symptoms. Increasing severity of alcohol use is associated with more risk for suicide ideation and suicide attempts.^31^ Notably, previous evidence suggests that alcohol is more related with the transformation from suicidal ideation to suicide attempt, possibly mediated by increased impulsivity.^12^ Thus, comorbidity with alcohol disorder may further augment the chance of suicide among individuals who began experiencing suicidal ideation during the pandemic.

Contrary to our hypothesis, we did not find an association between chronic diseases and the incidence of suicidal ideation. Physical illness is a known risk factor for suicide^28^ and self-reported physical health problems have been positively associated with suicidal ideation in older adults.^32^ In a previous analysis of our sample, we found that having more than one chronic disease during a specific wave of the COVID-19 pandemic was associated with anxiety disorders, but not with depressive disorders.^7^ Given the time span between the onset of the pandemic and the assessment period, individuals with pre-existing chronic disorders may exhibit increased worries and symptoms of anxiety. This may be particularly true since the pandemic was perceived as an urgent threat, and danger and threats are strongly associated with anxiety symptoms.^33^

We did not assess the subjective feeling of loneliness as an exposure during the pandemic, but we did use the objective condition of living alone, which had no significant influence on suicidal ideation incidence. Although both of these exposures are traditionally associated with suicidal ideation,^34^ loneliness is considered a more important risk factor.^35^ Indeed, a previous study conducted in Brazil during the COVID-19 pandemic found that loneliness but not social distancing variables were associated with suicidal ideation.^36^

An important strength of our study is that ELSA-Brasil is a well-defined cohort, reducing the risk of selection bias and consequently increasing the external validity and generalizability of our results. We had pre and peripandemic assessments of suicidal ideation using a well-validated clinical interview, the CIS-R. Other studies published by the same group showed that the prevalence rates of psychiatric diagnoses did not increase during the pandemic,^7^ contributing to greater comprehension of the psychopathology of this sample.

The limitations of this study should also be cited. First, our cohort is an occupational, rather than population-based sample, comprising public servants from USP who received their salary without any restriction. Thus, the findings should not be considered as nationally representative. Second, less than 50% of the sample answered the peripandemic online assessment, favoring younger individuals and with more digital literacy. This occurred in other cohorts that had similar or even higher rates of attrition during the pandemic.^37–39^ Third, the pandemic assessment was digital and self-administered, whilst the pre pandemic assessment was in person and administered by lay interviewers. Even so, an electronic and self-administered version of CIS-R has been previously validated.^19^

## Conclusion

In conclusion, our study found that individuals with prior mental health disorders and ACE were at greater risk of developing suicidal ideation during the pandemic. Despite the absence of an increase in suicide rates, the increase in suicidal ideation is an event that should be addressed by public health measures. These individuals should receive special attention regarding their suicide risk, since the effects of the pandemic will echo for many years still.
