# Supplementary material for: Incidence of suicidal ideation in a cohort of civil servants during the COVID-19 pandemic in Brazil: insights from the ELSA-Brasil Study
Source: Trends Psychiatry Psychother. 2024 Oct 28;46:e20230701. doi: 10.47626/2237-6089-2023-0701 (PMC11565249; doi:10.47626/2237-6089-2023-0701)
Supplement: Supplementary file 1 [file 2238-0019-trends-46-e20230701-suppl1.pdf]

http://doi.org/10.47626/2237-6089-2023-0701
Incidence of suicidal ideation in a cohort of civil servants during the COVID-19 pandemic in Brazil: insights from the ELSA-Brasil Study - Bacchi et al.

Table S1 - Comparison of responders and non-responders of wave-covid

	Overall (n = 4,297)	Non-responders (n = 2,180)	Responders (n = 2,117)	p-value
	n (%)	n (%)	n (%)	
Socio-economic				
Age (mean ± SD)	63.2 ± 8.8	64.1 ± 8.9	62.3 ± 8.4	< 0.001
Educational level				< 0.001
Bellow high school	275 (6.4)	252 (11.6)	23 (1.1)	
High school	309 (7.2)	260 (11.9)	49 (2.3)	
Incomplete college	1,719 (40.0)	931 (42.7)	788 (37.2)	
University degree	1,994 (46.4)	737 (33.8)	1,257 (59.4)	
Gender				< 0.001
Male	1,914 (44.5)	1,030 (47.2)	884 (41.8)	
Female	2,383 (55.5)	1,150 (52.8)	1,233 (58.2)	
Self-reported ethnicity				< 0.001
Black	580 (13.7)	356 (16.6)	224 (10.7)	
Mixed (brown)	889 (21.0)	528 (24.6)	361 (17.3)	
White	2,538 (59.9)	1,144 (53.2)	1,394 (66.7)	
Yellow	191 (4.5)	89 (4.1)	102 (4.9)	
Indigenous	41 (1.0)	32 (1.5)	9 (0.4)	
CIS-R diagnoses				
Depressive disorders				0.093
0	4,126 (96.0)	2,082 (95.5)	2,044 (96.6)	
1	171 (4.0)	98 (4.5)	73 (3.4)	
Anxious disorders				0.002
0	3,637 (84.7)	1,809 (83.0)	1,828 (86.5)	
1	657 (15.3)	371 (17.0)	286 (13.5)	
Obsessive compulsive disorder				0.803
0	4,197 (97.7)	2,131 (97.8)	2,066 (97.6)	
1	100 (2.3)	49 (2.2)	51 (2.4)	
Common mental disorder				0.012
0	3,262 (75.9)	1,619 (74.3)	1,643 (77.6)	
1	1,035 (24.1)	561 (25.7)	474 (22.4)	

CIS-R = Clinical Interview Schedule-Revised; SD = standard deviation.
Comparison between responders and non-responders using *t* test for continuous variables and χ^2-tests for categorical variables. Socioeconomic data reported from wave 1 (age based on date of birth) and CIS-R scores from wave 3 (collected between 2016-2018) was used for comparisons.
